# Sonic Hedgehog signaling in oligodendrogenesis, myelination, demyelinating diseases, and remyelination

**DOI:** 10.4103/NRR.NRR-D-25-00005

**Published:** 2025-08-13

**Authors:** Miguel Marchena-Fernández, Cristina Sánchez-Camacho, Emma Muñoz-Sáez, Alba Macías-Castellano, Fernando de Castro Soubriet

**Affiliations:** Grupo de Neurobiología del Desarrollo (GNDe), Instituto Cajal (CSIC) Madrid, Spain; Facultad HM de Ciencias de la Salud de la UCJC, Universidad Camilo José Cela, Madrid, Spain; NeuroLab, Instituto de Investigación Sanitaria HM Hospitales, Madrid, Spain; Department of Genetics, Physiology and Microbiology, Faculty of Biological Sciences, Complutense University of Madrid, Madrid, Spain; Department of Biosciences, School of Health and Biomedical Sciences, Universidad Europea de Madrid, Villaviciosa de Odón, Madrid, Spain

The Hedgehog (HH) family includes Indian (IHH), Desert (DHH), and Sonic Hedgehog (SHH). Proteins of the HH family are distinguished by their function as morphogens, i.e., molecules that regulate the pattern of tissue development in accordance with concentration gradient. Data accumulated over the years clearly demonstrate that HH signaling is essential in myelination, particularly in the life cycle of the oligodendrocyte lineage. DHH is a key factor for Schwann cell function in the myelination of the peripheral nervous system and IHH is directly involved in the specification of oligodendrocyte precursor cells (OPCs) at least in the zebrafish. The most studied family member in central nervous system (CNS) myelination is SHH. SHH signaling has been identified as a crucial component in oligodendrocyte differentiation and remyelination (Russo et al., 2024).

SHH is synthetized as a 46 kDa precursor protein which undergoes an autoproteolytic cleavage producing the excision of 19 kDa amino-terminal region (HH-N) and SHH carboxy-terminal polypeptide. The HH-N domain binds cholesterol and palmitic acid producing a functional signaling molecule.

Mechanisms underlying the generation of oligodendrocytes, the only myelinating CNS cells in physiological conditions, involve the activity of SHH in the embryo. Both basic helix-loop-helix transcription factors Olig1 and Olig2, induced by SHH, are required for OPC specification from the ventral ventricular zone in the developing brain and spinal cord (Laouarem and Traiffort, 2018). SHH also induces the proliferation of OPCs in the embryo and promotes their directional migration by binding to their Patched-1 receptor. SHH-induced OPC migration and proliferation are impaired after the blockage of megalin (low-density lipoprotein receptor-related protein 2), a member of the low-density lipoprotein receptor family (Ortega et al., 2012). In this context, the megalin receptor transiently internalizes SHH in astrocytes. These astrocytes control the release of SHH and thus the amount of this molecule available to the OPCs (Ortega et al., 2012). SHH is essential for generating oligodendroglial cells in the postnatal forebrain. These SHH-dependent molecular mechanisms, involved in the spatiotemporal generation of the oligodendroglial lineage, are also essential for postnatal myelin production where two components of the SHH signaling cascade, smoothened and GLI1 can be highlighted.

**Figure 1:**
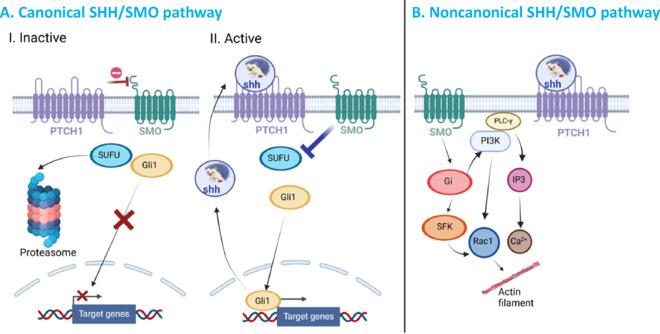
Diagram of the SHH/SMO signaling pathways. (A) Canonical SHH/SMO inactive (I) and active (II) pathway. (B) Noncanonical SHH/SMO pathway. Created with BioRender.com. Gi: Inhibitory G-protein; Gli: glioma-associated oncogene; IP3: inositol trisphosphate; PI3K: phosphatidylinositol 3-kinase; PLC: phospholipase C; PTCH1: pitched-1; Rac1: Ras-related C3 botulinum toxin substrate 1; SFK: Src family kinase; SHH: sonic hedgehog; SMO: smoothened protein; SUFU: suppressor of fused.

Smoothened (SMO) is a G protein-coupled receptor. The pitched-1 receptor, a twelve-pass transmembrane protein, constitutively inhibits SMO in the absence of SHH. During the canonical activation of the route, SHH binds to pitched-1, which derepresses SMO and induces the activation of the GLI transcription factors as downstream effectors (**Figure 1A**). Evidence points toward several noncanonical, GLI-independent pathways, operating via SRC family kinase, the small GTPases RAC1 and RHO-A, nuclear factor kappa B activation via PKC, and the Ca^2+^-AMPK axis (Teperino et al., 2012; **Figure 1B**). Important questions remain about the activity and regulation of the signal transducer SMO, relevant for developmental and therapeutic implications of the SHH signaling pathway in numerous disorders (see below).

Despite the proliferation of data on SHH, SMO, and intracellular signal transduction pathways, numerous inquiries remain unresolved. Research is required on the SHH/SMO pathway for the maturation of oligodendrocytes, and how they acquire the capacity to myelinate. Some researchers have indicated that an augmentation in SHH/SMO signaling may be a prerequisite for the maturation of oligodendrocytes to the final stage of development (Del Giovane and Ragnini-Wilson, 2018). Xu et al. (2020) suggest the opposite, that SHH/SMO signaling must decrease for the oligodendrocyte to complete differentiation.

**Figure 2:**
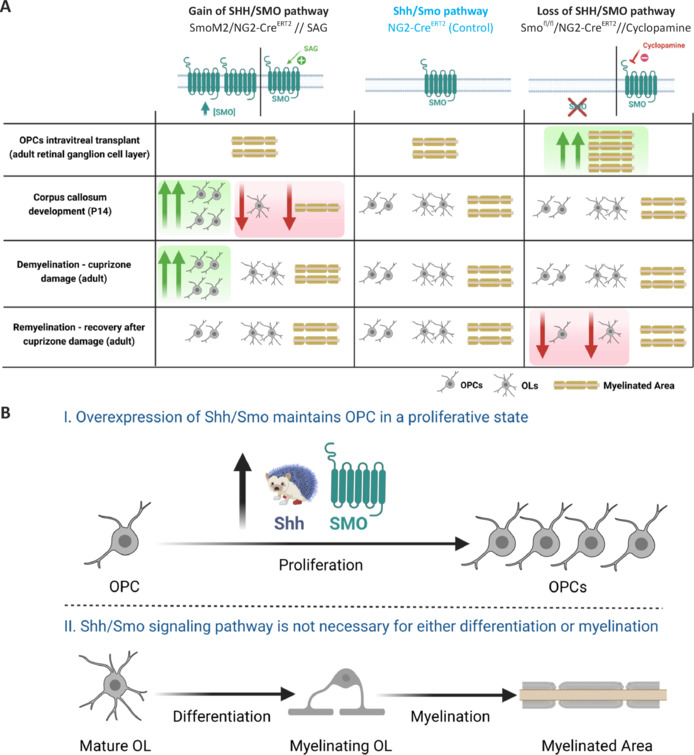
SHH signaling in oligodendrogenesis, myelination, demyelinating diseases, and remyelination processes. (A) Result summary modified from Nocera et al. (2024). In this work, the authors propose four different approaches. The first one is a naïve scenario for myelination which is the transplantation of OPCs intravitreally in adult mice, the second is a developmental scenario for myelination in the corpus callosum in P14 mice, the third, a cuprizone-induced demyelination in adult mice and the last scenario, a remyelination situation after demyelination with cuprizone. (B) Concluding remarks extracted from Nocera et al. (2024). (I) The activation of SHH/SMO is sufficient to maintain oligodendrocyte precursor cells in an undifferentiated state and (II) this signaling pathway is not necessary for myelin formation and (re)myelination. Created with BioRender.com. Cre: Cre recombinase; NG2: nerve/glia antigen-2; OL: oligodendrocyte; OPC: oligodendrocyte precursor cell; Shh: sonic hedgehog; Smo: Smoothened.

The recent study by Nocera et al. (2024) provides new insight into the role of SHH/SMO in maintaining OPCs in an undifferentiated state with the capacity to proliferate. It does not, however, play a direct role in the final differentiation to form myelin. The constitutive overactivation of SMO in the SmoM2/NG2-Cre^ERT2^ mice significantly increased the numbers of OPCs and decreased oligodendrocyte differentiation (including a smaller number of mature oligodendrocytes) during postnatal development (**Figure 2A**). The developmental myelination of the corpus callosum was not affected after the selective genetic ablation of SHH function in the Smo^fl/fl^/NG2-Cre^ERT2^ postnatal mice. The intravitreal segment of retinal ganglion cell axons provides a robust framework for corroborating these data. It is naturally lacking in oligodendrocytes (and myelin) because OPCs stop migration along the optic nerve during development and do not reach the interior of the eyeball. Adjacent to the intraocular segment of the retinal ganglion cell axons, the intravitreal space provides a physiological experimental paradigm devoid of the features that characterize the processes of development, demyelination, or remyelination. Even if a neurons-plus-oligodendrocytes co-culture is meticulously designed, this invariably is an artificial experimental framework. Intravitreal injection of OPCs genetically lacking the SHH/SMO pathway did not prevent the myelination of these axons. This process is even more significant than after injecting OPCs from control mice (Nocera et al., 2024). The effect is replicated when an inhibitor (cyclopamine) or an activator (SAG) of the SHH/SMO pathway is injected in conjunction with OPCs derived from control mice. Myelination of the intravitreal segment of retinal ganglion cell axons is significantly greater when OPCs are co-injected with cyclopamine than when they are co-injected with SAG or saline (Nocera et al., 2024). These data demonstrate the relevance of the activation of the SHH/SMO signaling pathway to favor the generation of OPCs as opposed to myelin formation during physiological CNS development. Immature oligodendrocytes do not require SHH/SMO activity to reach the end of their maturation process and to acquire the ability to myelinate axons (**Figure 2B**).

Given the role of SHH and SMO in the oligodendrocyte life cycle, both are important factors when considering demyelinating diseases such as multiple sclerosis and leukodystrophies. It can thus be predicted that both SHH and SMO will become the focus of attention in the design of treatments for these diseases.

A study carried out on models of active demyelination of the corpus callosum induced with cuprizone have demonstrated that the upregulation of the SHH/SMO pathway results in an increase of oligodendrocytic lineage cells (Nocera et al., 2024). When assessing the remyelination process 2 weeks after cessation of cuprizone intake, it was found that the loss of the SHH/SMO pathway during this time resulted in a smaller population of oligodendrocyte lineage cells (Nocera et al., 2024; **Figure 2A**).

These results and those described during the development and myelination of retinal axons open up the possibility of using SHH/SMO “switches” in the treatment of multiple sclerosis. The pathway would be “turned on” to increase the number of OPCs and “turned off” to promote oligodendrocyte maturation and myelination. This “switch” could be turned on by some of the molecules reported in the literature as agonists of the SHH/SMO pathway in different experimental models such as SAG (Smoothened agonist), GSA-10, clobetasol, and halcinonide (Fang et al., 2022). New molecules generated to minimize possible side effects or contraindications could be added to this list.

There is still a long way to go to ensure the success of strategies that focus on SHH/SMO to treat demyelinating diseases. SHH levels are finely adjusted, and any imbalance can lead to pathologies such as the appearance of tumors. There is a need for detailed research on the molecular “environment” in which the demyelination and remyelination processes occur, the interaction of the SHH/SMO pathway with other concomitant pathways during these processes, and how to experiment with SHH levels without complications.

We must not forget the rest of the elements involved in the SHH-dependent routes. A previous study in downstream components in the SHH/SMO pathway has demonstrated that Gli1 inhibition increases the recruitment of neural stem cells from the subventricular zone and their differentiation to oligodendrocytes. This leads to a functional improvement in a model of relapsing-remitting experimental autoimmune encephalomyelitis (Samanta et al., 2015).

Intervention on the SHH/SMO pathway may benefit not only patients with multiple sclerosis, but also those affected by leukodystrophies. Leukodystrophies are a panoply of rare progressive disorders affecting CNS myelin and usually lead to premature death. The role of SHH or the SHH/SMO pathway in the pathogenesis of leukodystrophies is still to be discovered (Chaudhary et al., 2024). Although anomaly has been observed in the SHH pathway in vanishing white matter leukodystrophy, another signaling route is preferred to target the illness, where Sigma-1 receptor agonists are employed when first attempting to treat the disease (Atzmon et al., 2018). The use of the SMO agonist SAG1 does not affect the neurons in 4H leukodystrophy or in their myelination *in vitro* (Dooves et al., 2023). The effects of SHH/SMO reported by Nocera et al. (2024) suggest that this signaling pathway deserves careful attention, especially in the case of rare leukodystrophies where anomalies in OPC lineage would be more relevant for pathogenesis.

In conclusion, a better understanding of the role of the SHH/SMO pathway in the oligodendrocytic lineage opens a wide range of possibilities in our fight against multiple sclerosis and leukodystrophies. The prospect of curing or even improving patient prognosis most certainly justifies efforts to delve into this exciting field of research.

*This work was supported by Spanish government (PID2022-143110OB-IOO) and Merck Foundation (20234599) grants (to FdeCS) and Univesidad Camilo Jose Cela (CEIDI_VI_08_06_XI_EMYDEM) grant (to MMF).*
